# Assessment of glomerular morphological patterns by deep learning algorithms

**DOI:** 10.1007/s40620-021-01221-9

**Published:** 2022-01-04

**Authors:** Cleo-Aron Weis, Jan Niklas Bindzus, Jonas Voigt, Marlen Runz, Svetlana Hertjens, Matthias M. Gaida, Zoran V. Popovic, Stefan Porubsky

**Affiliations:** 1 Institute of Pathology, University Medical Centre Mannheim, University of Heidelberg, 68167 Mannheim, Germany; 2 Mannheim Institute for Intelligent Systems in Medicine, University Medical Centre Mannheim, University of Heidelberg, Mannheim, Germany; 3 Institute of Medical Statistics and Biometry, Medical Faculty Mannheim, University of Heidelberg, Mannheim, Germany; 4 Institute of Pathology, University Medical Center of the Johannes Gutenberg University Mainz, Langenbeckstrasse 1, 55131 Mainz, Germany

**Keywords:** Machine learning, CNN, Classification, Glomerular change pattern

## Abstract

**Background:**

Compilation of different morphological lesion signatures is characteristic of renal pathology. Previous studies have documented the potential value of artificial intelligence (AI) in recognizing relatively clear-cut glomerular structures and patterns, such as segmental or global sclerosis or mesangial hypercellularity. This study aimed to test the capacity of deep learning algorithms to recognize complex glomerular structural changes that reflect common diagnostic dilemmas in nephropathology.

**Methods:**

For this purpose, we defined nine classes of glomerular morphological patterns and trained twelve convolutional neuronal network (CNN) models on these. The two-step training process was done on a first dataset defined by an expert nephropathologist (12,253 images) and a second consensus dataset (11,142 images) defined by three experts in the field.

**Results:**

The efficacy of CNN training was evaluated using another set with 180 consensus images, showing convincingly good classification results (kappa-values 0.838–0.938).

Furthermore, we elucidated the image areas decisive for CNN-based decision making by class activation maps. Finally, we demonstrated that the algorithm could decipher glomerular disease patterns coinciding in a single glomerulus (e.g. necrosis along with mesangial and endocapillary hypercellularity).

**Conclusions:**

In summary, our model, focusing on glomerular lesions detectable by conventional microscopy, is the first *sui generis* to deploy deep learning as a reliable and promising tool in recognition of even discrete and/or overlapping morphological changes. Our results provide a stimulus for ongoing projects that integrate further input levels next to morphology (such as immunohistochemistry, electron microscopy, and clinical information) to develop a novel tool applicable for routine diagnostic nephropathology.

**Supplementary Information:**

The online version contains supplementary material available at 10.1007/s40620-021-01221-9.

## Introduction

*Artificial intelligence* is an umbrella term encompassing techniques that enable machines to mimic human intelligence. *Machine learning* is a subset of artificial intelligence that refers to algorithms capable of learning without being explicitly programmed. Here, amongst others, so-called *convolutional neural networks* (CNNs) are implemented to independently ‘comprehend’ characteristic features in large image datasets. Recent substantial progress in image visualization and computation has made CNN-based machine learning an affordable approach that is of utmost interest for diagnostic image-based disciplines, particularly radiology and pathology [1–3]. Due to the plethora of sometimes overlapping clinical and histological disease patterns, nephropathology represents a challenging discipline. This holds true also for the evaluation of histology sections from experiments with kidney diseases in animal models. Here, maybe even more than in human samples, a higher degree of automation and standardization is needed. Algorithms for segmentation of healthy kidney parenchyma have been previously successfully developed [4, 5]. Similarly, computational morphologic analyses of diabetic nephropathy, mesangial proliferation, and IgA-Nephropathy pattern have been published [6–9]. However, until now, no CNN-based approach that simultaneously deals with various glomerular lesions and that can discern them from unaffected glomeruli has been reported.

A wide variety of glomerular lesions linked to specific kidney diseases have been well characterized [10]. In addition, many kidney diseases present a broad range of morphological alterations, which in sum reflect the morphologic and pathophysiologic complexity of glomerulopathies [10] (Sup. Figure 1). Maybe the most extreme example for this diagnostic complexity is the classification of lupus nephritis into different classes, each exhibiting a characteristic, however not pathognomonic, morphological signature [10–13]. Inline, other diagnostic entities may demonstrate a substantial variation in their morphology patterns that frequently reflect their clinical behavior (for instance, IgA nephropathy may also show an endocapillary in addition to the mesangial hypercellularity and necrosis at the same time). In this study, CNN models were trained to classify different glomerular lesions in a way that imitates the education of pathology residents to discern disease patterns before making the diagnosis [10–12]. Our approach was based on the digitalization of slides from the routine diagnostics and isolation of glomeruli. We demonstrate that it can successfully identify the glomerular lesions and recognize more than one disease pattern when confronted with complex glomerular changes.

## Methods

### Patient specimen and raw data generation

Periodic acid–Schiff (PAS) stained formalin-fixed paraffin-embedded tissue was retrieved (Institute of Pathology, Medical Faculty Mannheim, Heidelberg University & Institute of Pathology, University Medical Center of the Johannes Gutenberg University Mainz) and used in an anonymized way. The data collection and all experiments were conducted in accordance with a vote of the ethics commission II of the Heidelberg University (vote 2020-847R).

### Data management and analysis

Image processing (glomerulus segmentation, image cropping) and preprocessing were performed (image data arrangement) in the MATLAB environment (MATLAB (R2017a). Machine learning was performed in Python with PyTorch 14, 15 as described in the corresponding sections.

### Defining the patterns of glomerular changes

**Fig. 1:**
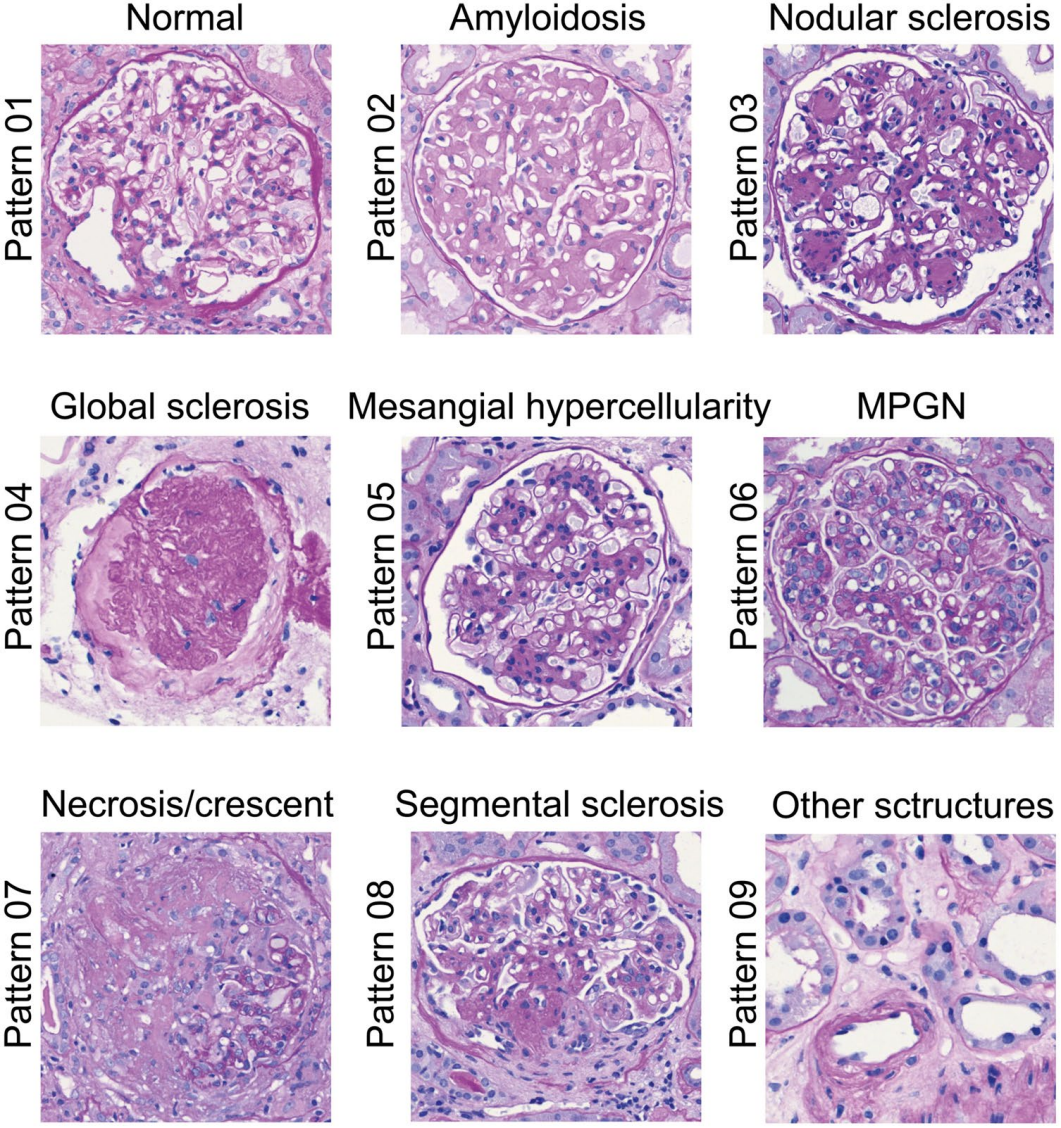
Paradigmatic patterns of glomerular diseases. In terms of conventional morphology, the fundamental glomerular changes were attributed to 9 patterns (Nrs. 01-09). Extraglomerular structures were labeled as ‘default’ pattern 09. MPGN, membranoproliferative glomerulonephritis

We first categorized the basal morphologic patterns of glomerular alterations for our CNN-model-based approach: normal glomerulus (pattern 01), amyloidosis (pattern 02), nodular sclerosis (pattern 03), global sclerosis (pattern 04), mesangial hypercellularity (pattern 05), mesangioproliferative glomerulonephritis (MPGN) (pattern 06), necrosis/crescent (pattern 07), and segmental sclerosis (pattern 08) (Fig. 1 and Sup. Figure 1). Extraglomerular structures that should offer an “exit strategy” for the models were labeled as pattern 09 (Fig. 1 and Sup. Figure 1–2). The categories represent prototypical glomerular changes in terms of light microscopy and the experiments do not rely on any ancillary studies that would have been implemented in routine diagnostics (such as immunohistochemical, immunofluorescence, or ultrastructural investigations). For example the typical pattern “amyloidosis” with its acellular, weakly PAS-positive depositions needs further investigations such as Congo-red stain (or EM) to establish the diagnosis and to exclude its mimics (such as fibrillary glomerulopathy).

### Creation of three datasets for training, validation, and testing

**Fig. 2:**
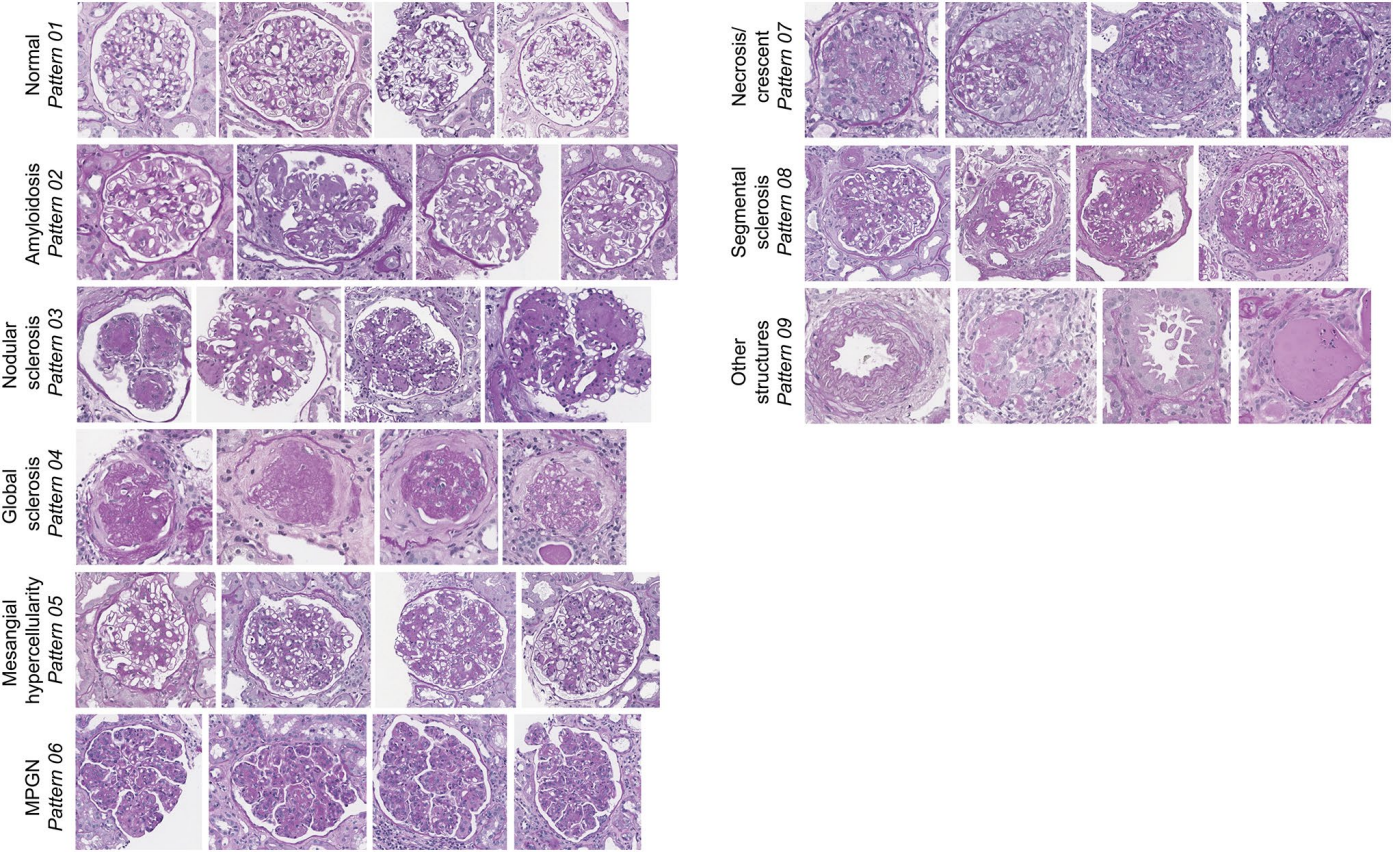
Examples of processed images. For each pattern, four examples from the image database are shown. The different image proportions are given by different bounding boxes reflecting the variable glomerular shape and size in the sections

We digitalized PAS stained slides of kidney biopsies from routine diagnostics from two independent institutions (Institute of Pathology Mannheim and Institute of Pathology Mainz). In total, three datasets (datasets #1 and #2 for training and validation and dataset #3 for testing only) were defined (Sup. Figure 2):

Dataset #1: To foster the collection of glomeruli from the above-mentioned scanned cases, we implemented a glomerulus detection RCNN-based object detection tool (as published by Kawazone et al. [16]). This tool automatically cropped all glomeruli per whole slide image. In the next step, an expert nephropathologist (SP) assigned a total of 12,253 images of cropped glomeruli with a single morphological pattern to the predefined patterns (Fig. 2) sorting out incomplete glomeruli or images with tissue artifacts. The numbers per pattern range from 70 for pattern 06 (MPGN) to 4385 for pattern 01 (normal glomerulus) (Sup. Figure 4 A1-2).

Datasets #2–3: A total of 11,142 newly cropped glomeruli were assigned to the predefined patterns in the consensus of three independent nephropathologists (SP, ZVP, MMG). The numbers per pattern range from 46 for pattern 06 (MPGN) to 4091 for pattern 01 (normal glomerulus) (Sup. Figure 4 B1-2). From this database, 20 images per pattern were chosen as the test set (hereafter dataset #3) and kept away until testing the models (Sup. Figure 4C). The remaining images (hereafter dataset #2) were used to re-train and validate the models pre-trained on dataset #1. All three nephropathologists performed their categorization of the images prior to the learning and testing phase, thus, not being influenced by any results of the CNN algorithm.

### CNN-training

Several published classification models or versions of published models (AlexNet [17], VGG [18], ResNet [19], Densenet [20], Squeeze net [21], and Inception net [22]) were trained (Sup. Figure 3). By using the repository hiddenlayer the architecture of the herein applied models can be visualized (as done in the folder ModelPlots in the according GitHub-repository cited below).’

The image size was set to 224 × 224 pixel for all models except the Inception net, where it was set to 299 × 299 pixel. As optimizer we used a stochastic gradient descent [23, 24]. The initial learning rate was 10^–3^. We used a learning rate decay of 0.1 every 7 epochs. All models were trained for 50 epochs. The loss function was cross entropy loss.

All calculations were performed on a GPU (Graphics Processing Unit) (NVidia TitanXP).

In a typical transfer learning setting a complex, published model, which initially was trained on a large dataset (e.g. the cityscape dataset), is re-trained on a smaller dataset. The idea behind this is that complex models may not converge if trained only on a small dataset. By using a pre-trained model, this model has already learned many features and usually only needs to re-learn how to map the features on the new classes. Therefore, in a typical transfer learning setting only the last layers of a CNN model are re-trained [25–27]. However, in our model, re-training of the last layer solely did not lead to a convergence of the loss, likely due to a specific image composition of the kidney tissue [28–30] in contrast to large image datasets usually used for training. For instance, histological images have quite a different color composition in contrast to cityscapes.

### Statistical analysis

Statistical analysis was performed in R version 3.2.4 [31]. Kappa coefficients were calculated to assess the degree of agreement for binary factors. In the case of more than two raters, the Fleiss' kappa was calculated. As a measure for classification quality, the accuracy and the confusion matrix are presented.

### Code and data availability

The according code is available on GitHub: https://github.com/catweis/Assessment-of-glomerular-morphological-patterns-by-deep-learning.

The trained models and the test dataset are available on HeiData: https://heidata.uni-heidelberg.de/privateurl.xhtml?token=6f166ca5-c48a-4943-86c7-4b35c88dc879.

## Results

### Training and validation of different convolutional neuronal network (CNN) classification models

We used different published classification models [14, 15] and the aforementioned dataset #1 (defined by one expert pathologist) and dataset #2 (consensus of three expert pathologists) to classify glomerular disease patterns. In addition, we aimed to compare the performance of different convolutional neuronal networks CNNs in terms of accuracy. To this end, several versions of AlexNet [17], VGG [18], ResNet [19], Densenet [20], Squeeze net [21], and Inception net [22] were trained (Sup. Figure 2–3). Examples of the model input are shown in Fig. 2.

The classification quality for all networks was assessed based on the untrained part (20%) of datasets #1 and #2 used for validation purposes (hereinafter validation sets #1 and #2). No cross-validation approach was used; thus, the images within the validation sets are unseen. The accuracy and kappa value were calculated based on the resulting confusion matrices. The accuracy values ranged from 0.910 to 0.920 (validation set #1) and respectively 0.944–0.984 (validation set #2). The kappa values ranged from 0.884 to 0.933 (validation set #1) and respectively 0.927–0.979 (validation set #2). In summary, the ResNet-variant ResNet101 produced the best results for the validation sets (Table 1 and Fig. 2).

**Table 1:** CNN-model results of the validation dataset (each part of dataset #1 and #2)

Model	Accuracy	Kappa value
AlexNet [17]	0.910/0.944	0.884/0.927
squeeznet [21]	0.912/0.945	0.886/0.928
vgg11 [18]	0.932/0.963	0.912/0.951
vgg19 [18]	0.940/0.968	0.912/0.958
ResNet18 [19]	0.940/0.970	0.922/0.960
vgg16 [18]	0.933/0.970	0.913/0.960
ResNet34 [19]	0.954/0.975	0.940/0.967
ResNet50 [19]	0.949/0.977	0.934/0.970
inception [22]	0.947/0.980	0.930/0.973
densenet121 [20]	0.955/0.980	0.941/0.974
ResNet152 [19]	0.947/0.981	0.932/0.975
ResNet101 [19]	0.949/0.984	0.933/0.979

From dataset #1 (n = 2451 images) and from dataset #2 (n = 2267), each corresponding to 20%, are used for validation only. The table below shows the accuracy and kappa-values for different models, with the first value for dataset #1 and the second for dataset #2

In order to measure the classification accuracy for each pattern separately, Fleiss’ kappa-value was calculated in all trained models. Independently of the model used, the best accordance (i.e., highest kappa-values) could be observed for the normal glomerulus (pattern 01; 0.967), MPGN (pattern 06; 0.985), and other structures (pattern 09; 0.0971). In contrast, the values were lowest for mesangial hypercellularity (pattern 05; 0.863). All remaining kappa-values were in the range in between.

### Testing of the trained CNN-models

**Fig. 3:**
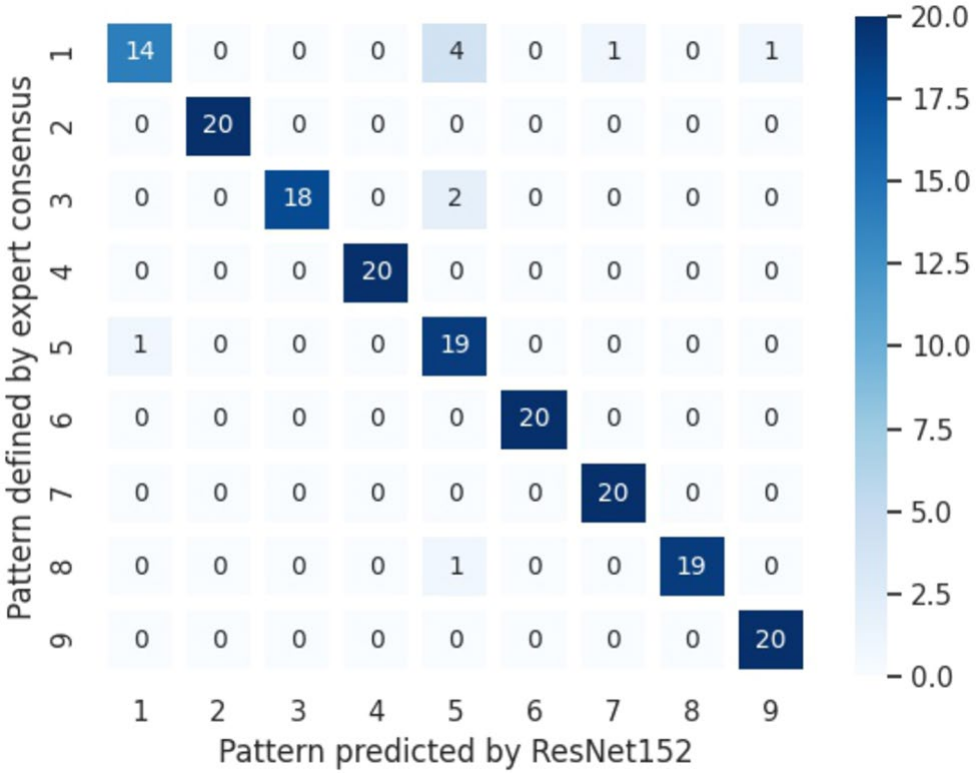
Performance of the CNN algorithm. Confusion matrix depicting the results of CNN-based glomerular categorization (by the ResNet 152) on the x-axis compared with the expert consensus on the y-axis. The herein analyzed test set contains 20 images per pattern. Here the accuracy was 0.944, and the kappa-value 0.938

The test dataset—dataset #3—contained 180 images assigned to the predefined patterns by consensus of three independent expert nephropathologists (MMG, SP, and ZVP). Of note, in contrast to the previously used validation sets, the test set was balanced with 20 images per pattern. The images were analyzed using all the models mentioned above, and the accuracy and kappa value were calculated based on the resulting confusion matrices. The accuracy values ranged from 0.856 to 0.944, and the kappa values ranged from 0.838 to 0.938. In summary, the ResNet-variant ResNet152 (Fig. 3) produced the best results for the test set (Table 2). The Fleiss’ kappa values reached the best accordance for MPGN (pattern 06; 0.985) and the worst for mesangial hypercellularity (pattern 05; 0.756).

**Table 2:** Results for the test dataset (dataset #3)

Model	Accuracy	Kappa value
AlexNet [17]	0.856	0.838
squeeznet [21]	0.861	0.844
ResNet50 [19]	0.900	0.888
ResNet101 [19]	0.900	0.888
vgg11 [18]	0.911	0.900
vgg19 [18]	0.911	0.900
ResNet18 [19]	0.917	0.906
ResNet34 [19]	0.928	0.919
densenet121 [20]	0.928	0.919
inception [22]	0.928	0.919
vgg16 [18]	0.939	0.931
ResNet152 [19]	0.944	0.938

Dataset #3 comprises n = 180 images that were categorized by three nephropathologists (MMG, SP, and ZVP). For the trained models, the accuracy and the kappa value were calculated

### Identification of the image areas decisive for the CNN-decision making

**Fig. 4:**
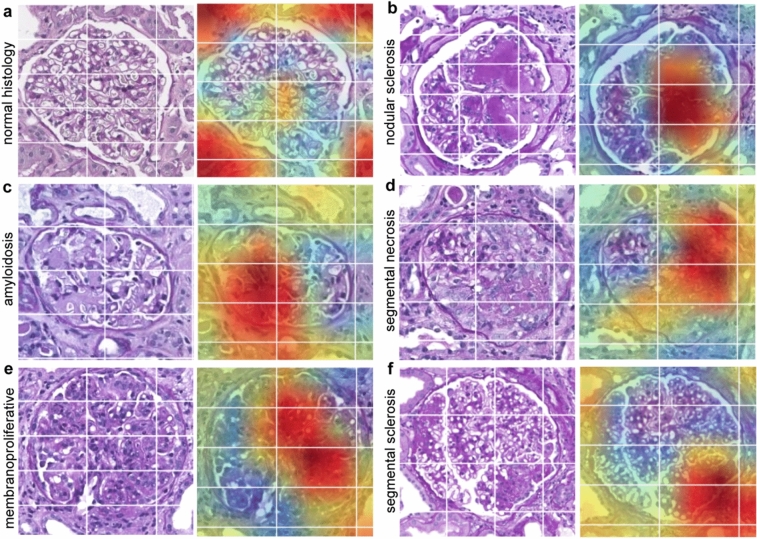
Class activation maps (CAM) visualizing the decision-relevant image parts. In the trained CNN model, CAM was used to detect the image regions responsible for the strongest activation of the corresponding class. Shown are selected glomerular disease patterns along with a heat map visualizing areas in dark red that were most decisive for the class activation. In **b**–**f**, these areas are congruent with the foci of morphological alteration. In panel **a** (normal glomerulus), there was no pathology, and therefore no activation of the model trained on pathology classification

In order to elucidate the CNN-based classification process, we strived to identify which aspects of the image are essential for decision making. We implemented two different approaches:

1. First, we used a class activation map (CAM) to produce a heat map for the network's highest activation, leading to the correct class assignment for every image [25]. We applied a free-available Pytorch-implementation from GitHub [26]. At one end of the spectrum, in the setting of normal glomerulus (pattern 01), the model mainly focused on the edges of the image, where small parts of the tubulointerstitium and the blood vessels are shown (Fig. 4A), implying that recognition of normal glomerulus may be an indirect result of the exclusion of non-glomerular structures and incomplete glomeruli. On the other hand, in the case of amyloidosis (pattern 02), the heat maps showed that the model directly screened amyloid deposition within the glomerulus and, interestingly, in the interstitial blood vessels (in Fig. 4B). Also, in other patterns, the heat maps apparently directly highlighted the pathological regions within the image.
2. Second, we used a deep convolutional generative adversarial network (DCGAN) to produce example images for each class. The code was derived from the book by Hany and Walters [27] and is publicly available on GitHub [32]. The model consists of two networks: a generator learns to create synthetic image samples from a given distribution, and a discriminator being a classifier that distinguishes between authentic images from the given dataset and the fake ones created by the generator. Both networks are trained simultaneously in an adversarial process in which the generator tries to deceive the discriminator while the discriminator tries to identify the fake images. For normal glomeruli (pattern 01), this approach led to blurred but rather specific images, in the sense that the glomeruli were recognized as more or less physiological (Supp. Figure 5A). The images were also rather specific for global sclerosis (pattern 04) (Supp. Figure 5D). However, in both cases, the hereby-produced images were easily recognized as non-original due to their limited image quality. For the other patterns, the produced images were non-distinguishable. The network generated blurred images for all analyzed classes (Supp. Figure 5).

### Application of the trained model in diagnostic settings

In practical terms, a kidney biopsy can contain glomeruli that do not necessarily show the same disease pattern and are heterogeneously distributed; for example, a renal biopsy from a patient with nephrosclerosis may contain normal, segmentally, and globally sclerosed glomeruli. Similarly, a case of necrotizing IgA glomerulonephritis may show mesangial expansion, endocapillary proliferation, necrosis, as well as segmentally and globally sclerosed glomeruli.

Thus, to determine how the algorithm will deal with real biopsy cases, we manually cropped glomeruli from 46 diagnostic slides and assigned them to one of the previously defined disease patterns.

The trained ResNet152-model evaluated every cropped image for all cases and produced a binary output: the predicted value can be within (correct choice) or outside the expected range (false choice). The mean correct rate was 0.867 with a standard deviation of 0.169. Pattern 09 (other structures) as default pattern was among the wrong choices, the most frequent one. Pattern distributions per diagnosis and diagnosis group were plotted per case in a heat map (Supp. Figure 6). Some categories with a very characteristic morphological signature (like amyloidosis) tended to be assigned correctly, whereas other morphologically more similar patterns such as mesangial hypercellularity and nodular sclerosis showed poorer classification quality.

### Detection of more than one disease pattern in a single glomerulus

**Fig. 5:**
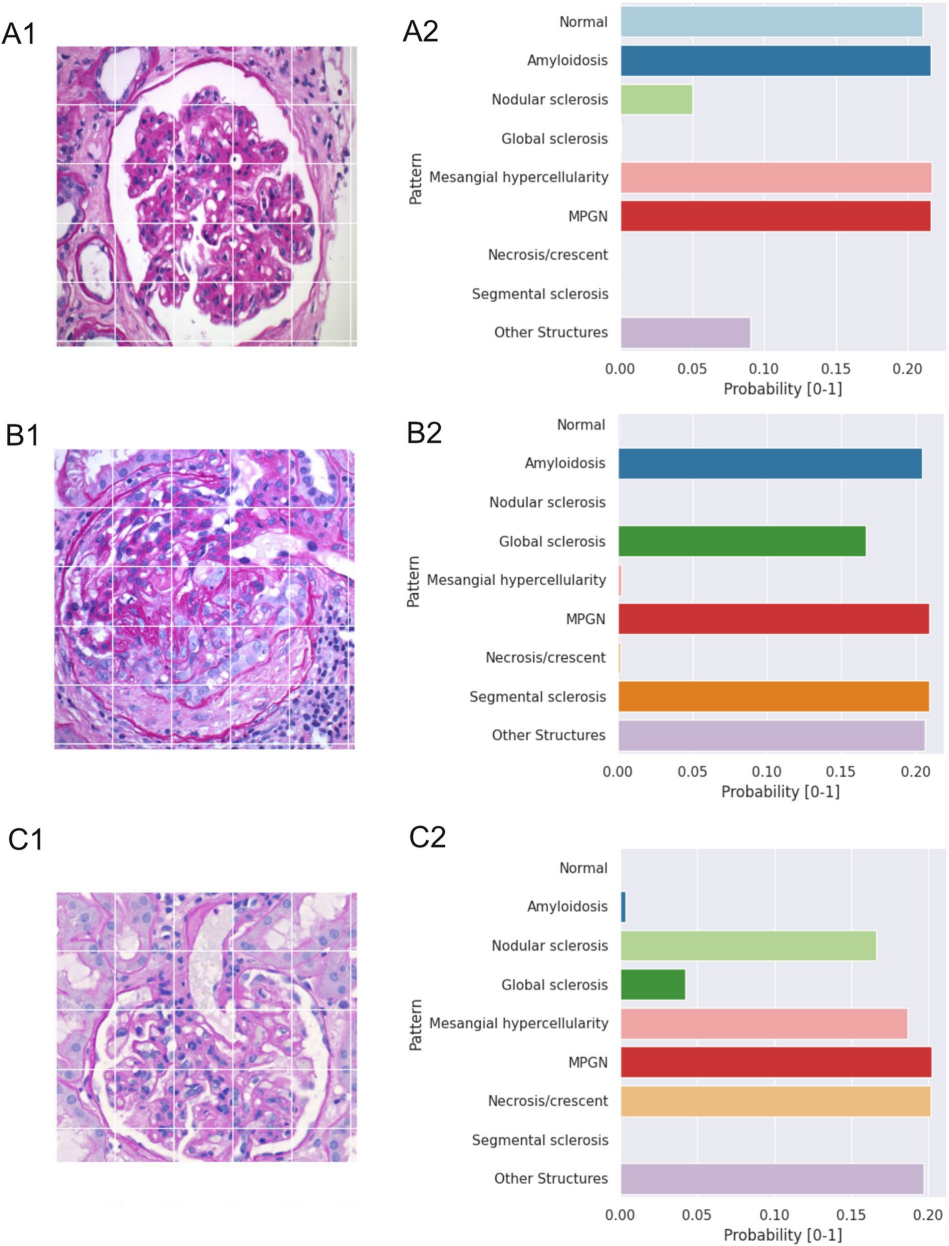
CNN-based algorithms are capable of recognizing several coincident disease patterns in one glomerulus in “real-life” images. Images of glomeruli with more than one disease pattern were taken from the daily diagnostic routine and subjected to CNN analysis. This generated probability values ranging from 0 to 1, reflecting the 'similarity’ to the nine classes for each image. **A** IgA-glomerulonephritis with endocapillary hypercellularity and incipient segmental sclerosis. **B** IgA-glomerulonephritis with extracapillary proliferation progressing to sclerosis that reaches the border between segmental and global. **C** MPGN with small extracapillary proliferation progressing to segmental sclerosis

A synchronous combination of different morphological patterns can occur both on the whole biopsy and on the single glomerulus level, for example, segmental necrosis in MPGN or segmental sclerosis in a glomerulus with mesangial hypercellularity. To test the model’s decision on such glomeruli with more than one pattern, we used new images of glomeruli with complex changes that cannot be attributed to any single category but show facets of several patterns in parallel. For every input image, the model produced a probability value for all nine classes ranging from 0 to 1. As shown in Fig. 5, the algorithm “recognizes” more than one disease pattern reflected by approximately equally high probabilities of the relevant patterns. Thus, in a case of IgA-glomerulonephritis with endocapillary hypercellularity and incipient segmental sclerosis (Fig. 5A), the 'mesangial hypercellularity’ and ‘segmental sclerosis’ were identified along with a similarity to the ‘MPGN’ pattern that reflects the endocapillary hypercellularity in this case. Of note, here and in most of the analyzed ambivalent cases, the basket-category 'other structures' received a relatively high rating—not surprising if appreciating that the training was performed on 'single patterned’ images. In the next step, we selected a more complex image from a case of IgA-nephropathy showing a combination of mesangial and extracapillary proliferation progressing to sclerosis (Fig. 5B). Our model correctly reflected the fact that the sclerosis reached the border between segmental and global (i.e. 50% of the glomerular area) by assigning approximately equal ratings for the patterns of segmental and global sclerosis. Indeed, as collapsed capillaries with extracapillary proliferation dominate here, discrete mesangial hypercellularity in this case was not recognized by the network. Interestingly, the homogeneous fibrous tissue “resembled” amyloidosis to some extent (Fig. 5B). In a third case of a MPGN with a small necrosis progressing to segmental sclerosis, we could observe accurate categorization of all the relevant, even discrete patterns (i.e. ‘MPGN’, ‘mesangial hypercellularity, ‘necrosis/crescent’ and ‘segmental sclerosis’).

## Discussion

In the present study, we investigated the potential value of machine learning in automatized recognition of common glomerular morphological changes. As quality parameter for the classification, we used the well-established kappa-value, a classical tool to measure inter-rater reliability and agreement for nominal (diagnostic) decisions by (human) experts. Importantly, we initially intended to exclude the influence of interobserver variability (as one of the hallmarks of nephropathology) and focus on the capacity of CNN to recognize (even complex, but) clear-cut patterns. Hence, our models were first trained on a dataset initially defined by one expert pathologist, in the next phase, they were re-trained on a second dataset defined based on a three-expert consensus and finally evaluated on a subpart of the second, three-pathologist-consensus dataset (Sup. Figure 2). Whereas calculation of the accuracy in our study provided an overall measurement for the classification quality, kappa statistics mirrored the agreement for every class. For the herein defined eight non-overlapping patterns of glomerular lesions and for a ninth evasion category, the trained CNN-models showed kappa-values from 0.884 (AlexNet-model) [14, 15, 17] to 0.979 (ResNet 101-model) [14, 15, 19] for the validation datasets, hence demonstrating good to very good accordance with the provided expert opinion. Furthermore, in a dedicated test set with images evaluated and selected by the consensus of three experts from two independent institutes, the kappa-values ranged as well from good to very good (0.838 for the AlexNet-model [14, 15, 17] to 0.938 for the ResNet 152-model [14, 15, 19]).

As expected, successful recognition of morphological patterns was not sufficient for an accurate assignment to the appropriate diagnosis in most cases (Sup. Figure 1). A typical example of a glomerular disease with a limited set of pathological changes is amyloidosis (pattern 02). Although the establishment of the diagnosis “amyloidosis” and the exclusion of its mimics (e.g. the rare fibrillary glomerulopathy) need further investigations (such as Congo-red-stain or electron microscopy), the amyloidosis-pattern is quite characteristic. Accordingly, the diagnosis of amyloidosis was recognized by the ResNet152 in the diagnostic test set with 100% accuracy. On the other hand, immune-complex glomerulonephritides like IgA glomerulonephritis, lupus nephritis with a spectrum of rather unspecific lesion patterns obviously require further input from immunohistochemical/immunofluorescence and ultrastructural studies as well as clinical data for the completion of the diagnostic process.

The comparison of the performance of our trained models with other classification models is hampered by several points: many popular image datasets (like CIFAR-10) do not correspond to our model regarding the image content since they contain roughly defined morphological classes (‘dog’, ‘sheep’, etc.) that are not comparable with rather delicate alterations of glomerular histology. Next, the data pool in our study comprised in total 23,395 images assigned to nine classes. As a comparison, broadly used datasets like the ImageNet 2012 classification dataset cover more than 1000 classes in more than 1 million images [33]. Furthermore, even if certain published datasets contain histological images (for instance, the Atlas of Digital Pathology dataset with a significantly larger image cluster—17,668 images grouped into 42 classes), the image content and the task definition (recognition of the histological tissue type) substantially differ from our model [34]. We focused on highly specific glomerular architecture and set the task to recognize proportionally discrete pathological changes.

Moreover, the comparison of our results to other published models is hampered by the differences regarding the underlying data and partially by the metrics employed for the classification quality. As generally accepted, we applied accuracy as an overall measure for the classification quality. Considering the above-mentioned work by Hosseini et al. [34] that reported an accuracy ≥ 0.95 for all used models, our results from a proportionally much smaller dataset (accuracy values 0.856–0.944 for the test set) are comparable. Nevertheless, dealing with a diagnostic problem, we also applied typical inter-rater statistical values like the kappa-value and Fleiss kappa-value, used only in a minority of papers. For instance, in a trained CNN-model for the classification of diabetic retinopathy in fundus images, Gyatri et al. [35] reported a kappa-value for multiclass classification of 0.994. The kappa-values for the multiclass problem of the nine patterns in our study were in the range 0.756–0.985. In summary, the herein tested models can distinguish between most of the patterns with very high accuracy.

Importantly, our models were first trained on a dataset initially defined by one expert pathologist, and in the next phase they were re-trained on a second dataset defined based on a three-expert consensus. Finally, the models were evaluated on a test set that is a subpart of the second, three-pathologist-consensus dataset. There, the kappa-values for all herein trained and tested models are in the range from good to very good.

The major challenge of glomerular pathology is that a single glomerulus can bear a combination of different disease patterns. This particular problem of overlapping tasks distinguishes our project from other approaches that trained CNNs to categorize tumors in mutually exclusive, rigorous categories like tumor type, microsatellite stability, or prognostic category [36, 37]. Here we could demonstrate that our CNN model dealt with complex cases derived from routine diagnostics effectively, and recognized several co-existing lesion patterns occurring in a single glomerulus.

It is important to stress that—similar to routine diagnostics—the recognition of a particular glomerular pattern does not equal the diagnosis which is the result of a complex process of considering ancillary tissue studies, clinical information, serological data and patient’s history. For example, this input is essential for the interpretation of mesangial hypercellularity that can be the manifestation of an IgA-glomerulonephritis, lupus nephritis or another connective-tissue disease. Similarly, the light-microscopic pattern “normal glomerulus” might still lead to the diagnosis of an early membranous glomerulonephritis or minimal-change glomerulopathy if the characteristic ultrastructural findings and proteinuria are present.

In summary, our model shows that artificial intelligence may be used as a reliable model in the up-front evaluation of kidney pathology in biopsy specimens. Moreover, the presented algorithm is capable of “recognizing” the affection of a single glomerulus by more than one disease pattern—a situation regularly encountered in diagnostic nephropathology. Further studies are warranted to integrate also the immunohistochemical and ultrastructural patterns together with clinical parameters (such as proteinuria, erythrocyturia, kidney function, and serum parameters) to boost the accuracy of digital learning in nephropathology and to present a valuable tool applicable in routine diagnostics in the future.

## Supplementary Information

Below is the link to the electronic supplementary material.

Sup. Fig. 1 Schematic presentation of the relation between morphological pattern and diagnoses. In nephropathology, there is a complex setting with different morphological patterns (represented by symbols) that are associated in different combinations and extends with disease entities (represented by letters.) (PNG 307 kb)

Sup. Fig. 2 : Flow chart for the model training and testing. PAS-stained kidney biopsies were digitized and glomeruli automatically (dataset #1) or manually (dataset #2-3) cropped. Subsequently, these glomeruli were assigned to the predefined patterns by one expert (SP dataset #2) or based on the consensus of three experts (SP, MMG, ZVP datasets #2-3). In this sorting step, artifacts etc. are removed. The consensus-based data set was decomposed into two parts: Dataset #2 was used for retraining the models, while Dataset #3 was used only for testing. (PNG 372 kb)

Sup. Fig. 3 : Black box-representation of the used models. In the present work, glomeruli were assigned to nine predefined patterns using a CNN model. The CNN model can be seen as a black box in which 12 different CNN models published by other groups were trained, validated and tested. (PNG 1058 kb)

Sup. Fig. 4 : Overview of the training, validation, and test sets. A: Database #1 encompassed 2,650 cases categorized by expert #1 and was split into a training and validation set with factors 0.75 and 0.25, respectively. B: Database #2 encompassed 234 images independently categorized by experts #1 and #2. This database was used for testing only. (PNG 688 kb)

Supp. Fig. 5 : Five examples of generative adversarial network results per defined pattern. Generative adversarial networks (GAN) combine two neural networks that can be used to produce example images from a trained CNN model. A-I show five example images trained for every class. (PNG 6398 kb)

Supp. Fig. 6 : Pattern distribution for a small heterogeneous cohort from routine diagnostics. For a small cohort composed of routine cases, all glomeruli per biopsy were cropped and subsequently analyzed. For these cases, a set of 12 diagnoses were defined: amyloidosis, diabetic nephropathy, ERSD (end-stage renal disease), FSGS (focal segmental glomerulosclerosis), IgA, membranous GN (glomerulonephritis), minimal change, MPGN (membranoproliferative glomerulonephritis), necrotizing GN (glomerulonephritis), nephrosclerosis, normal and SLE (systemic lupus erythematosus). The cases were arranged along the y-axis; thus, every row corresponds to one case. For every glomerulus, the main pattern was diagnosed by the ResNet152. The patterns are depicted on the x-axis. (PNG 217 kb)
