# Supplementary figures and images for: Assessment of glomerular morphological patterns by deep learning algorithms
Source: J Nephrol. 2022 Jan 4;35(2):417–27. doi: 10.1007/s40620-021-01221-9 (PMC8927010; doi:10.1007/s40620-021-01221-9)

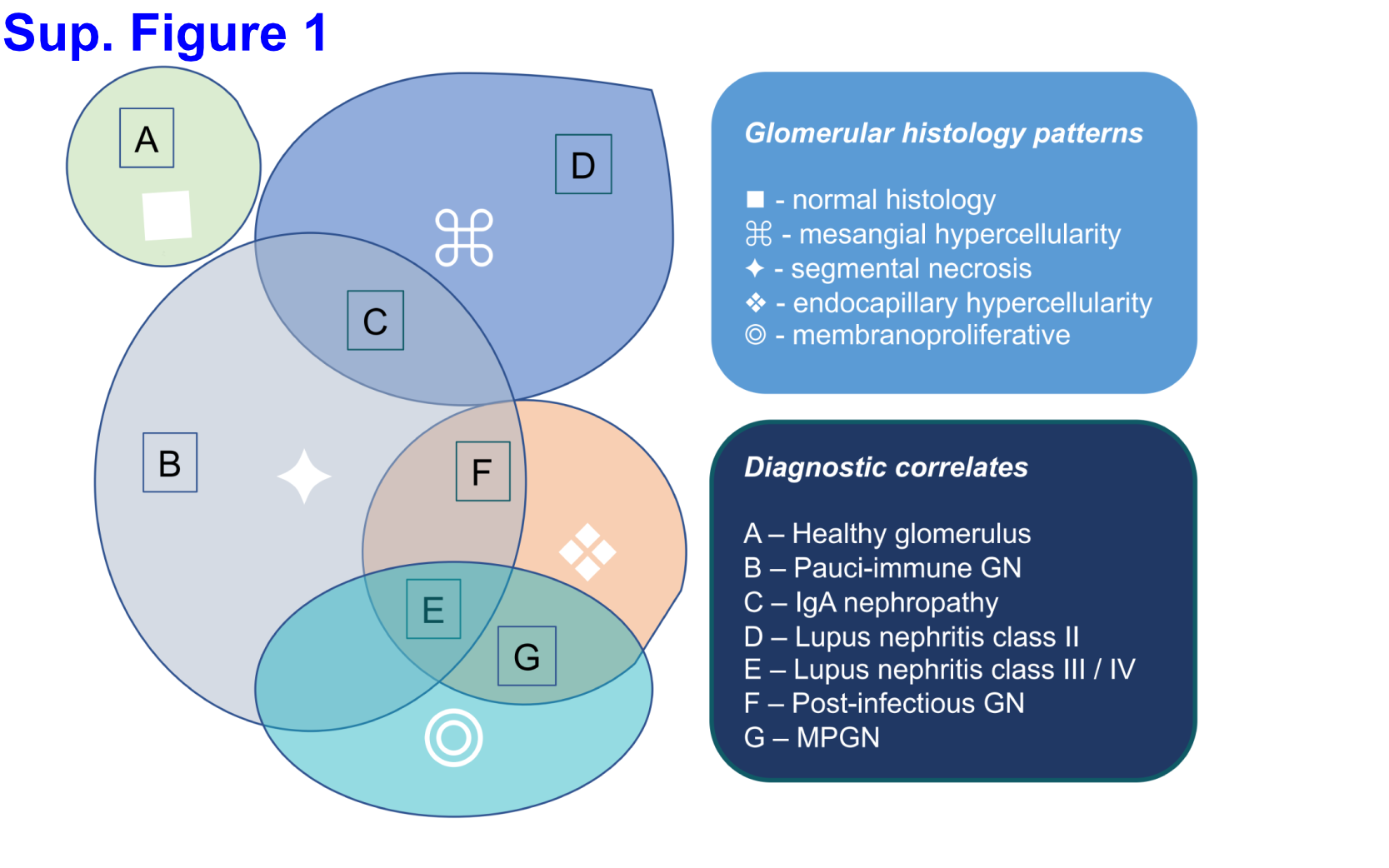

Supplement: Supplementary file 1 — Sup. Fig. 1 Schematic presentation of the relation between morphological pattern and diagnoses. In nephropathology, there is a complex setting with different morphological patterns (represented by symbols) that are associated in different combinations and extends with disease entities (represented by letters.) (PNG 307 kb) [file 40620_2021_1221_MOESM1_ESM.png]

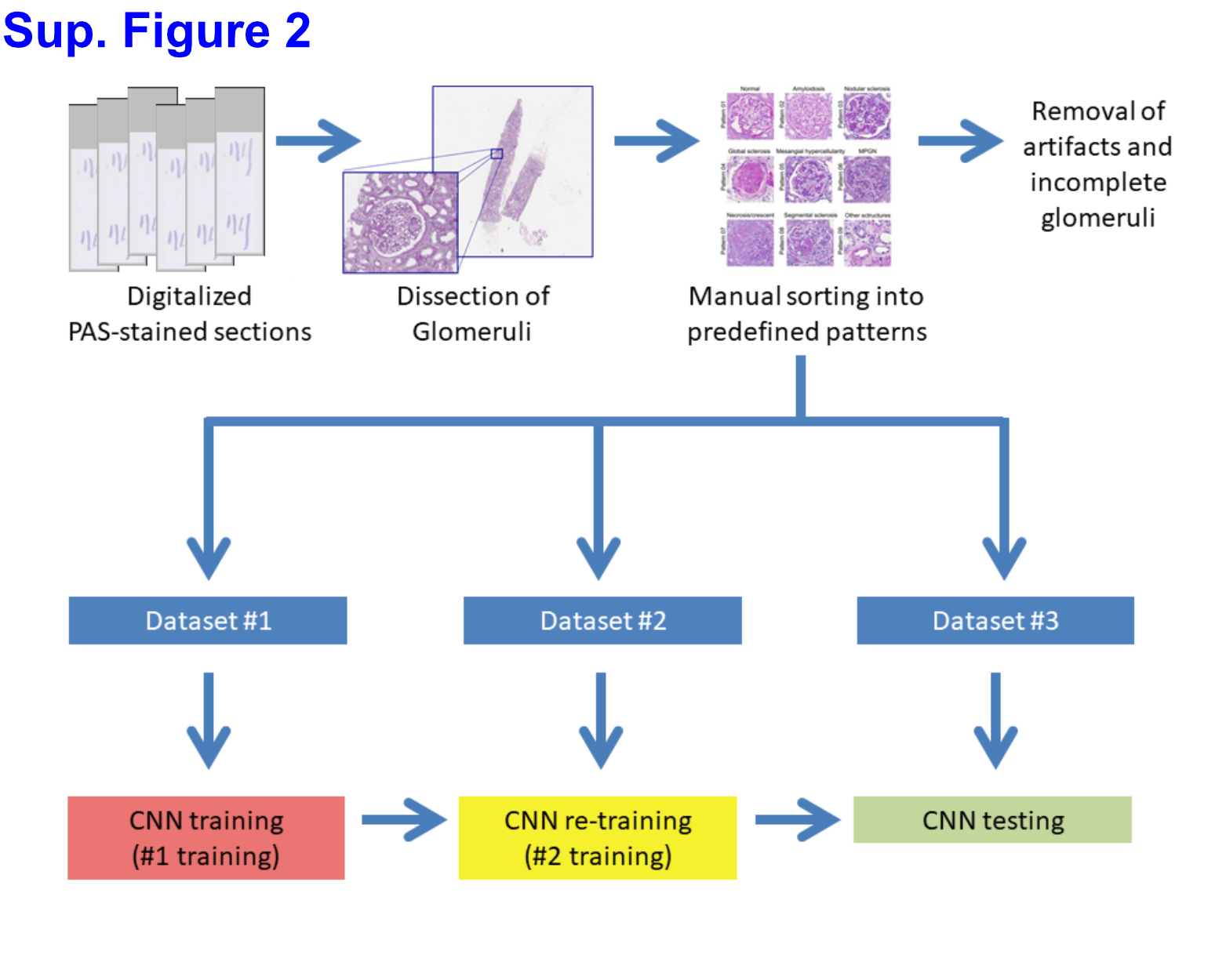

Supplement: Supplementary file 2 — Sup. Fig. 2 : Flow chart for the model training and testing. PAS-stained kidney biopsies were digitized and glomeruli automatically (dataset #1) or manually (dataset #2-3) cropped. Subsequently, these glomeruli were assigned to the predefined patterns by one expert (SP dataset #2) or based on the consensus of three experts (SP, MMG, ZVP datasets #2-3). In this sorting step, artifacts etc. are removed. The consensus-based data set was decomposed into two parts: Dataset #2 was used for retraining the models, while Dataset #3 was used only for testing. (PNG 372 kb) [file 40620_2021_1221_MOESM2_ESM.png]

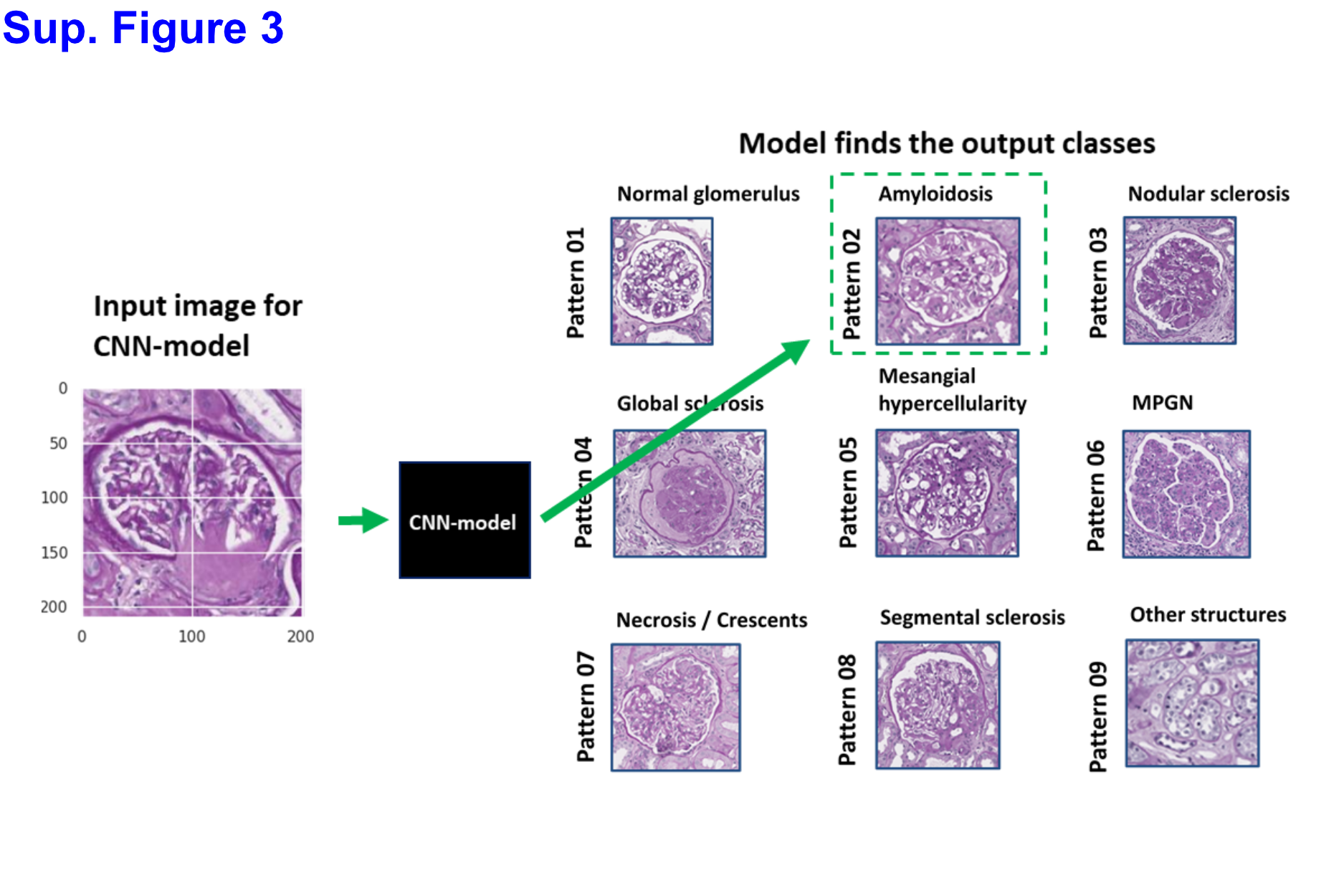

Supplement: Supplementary file 3 — Sup. Fig. 3 : Black box-representation of the used models. In the present work, glomeruli were assigned to nine predefined patterns using a CNN model. The CNN model can be seen as a black box in which 12 different CNN models published by other groups were trained, validated and tested. (PNG 1058 kb) [file 40620_2021_1221_MOESM3_ESM.png]

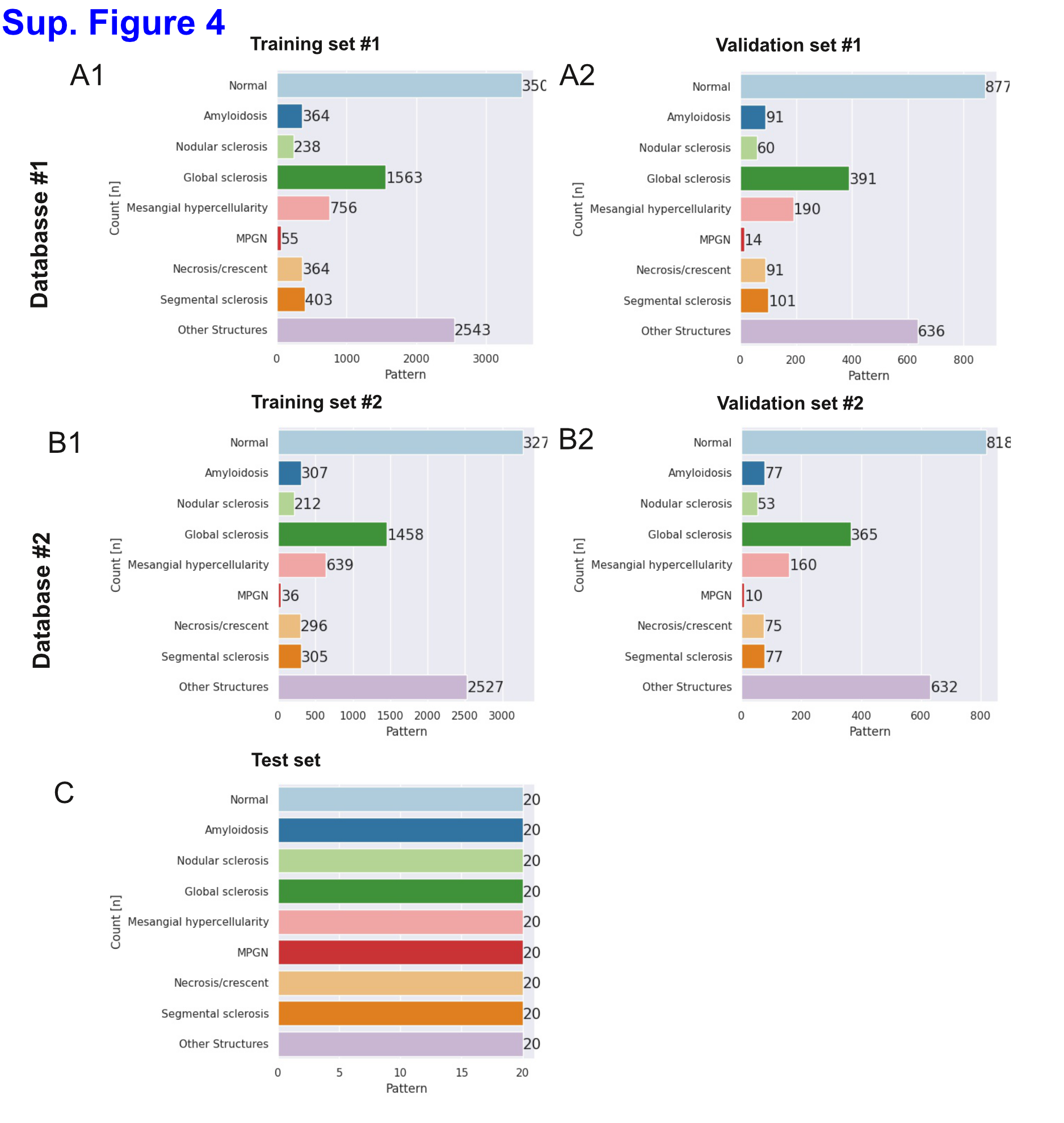

Supplement: Supplementary file 4 — Sup. Fig. 4 : Overview of the training, validation, and test sets. A: Database #1 encompassed 2,650 cases categorized by expert #1 and was split into a training and validation set with factors 0.75 and 0.25, respectively. B: Database #2 encompassed 234 images independently categorized by experts #1 and #2. This database was used for testing only. (PNG 688 kb) [file 40620_2021_1221_MOESM4_ESM.png]

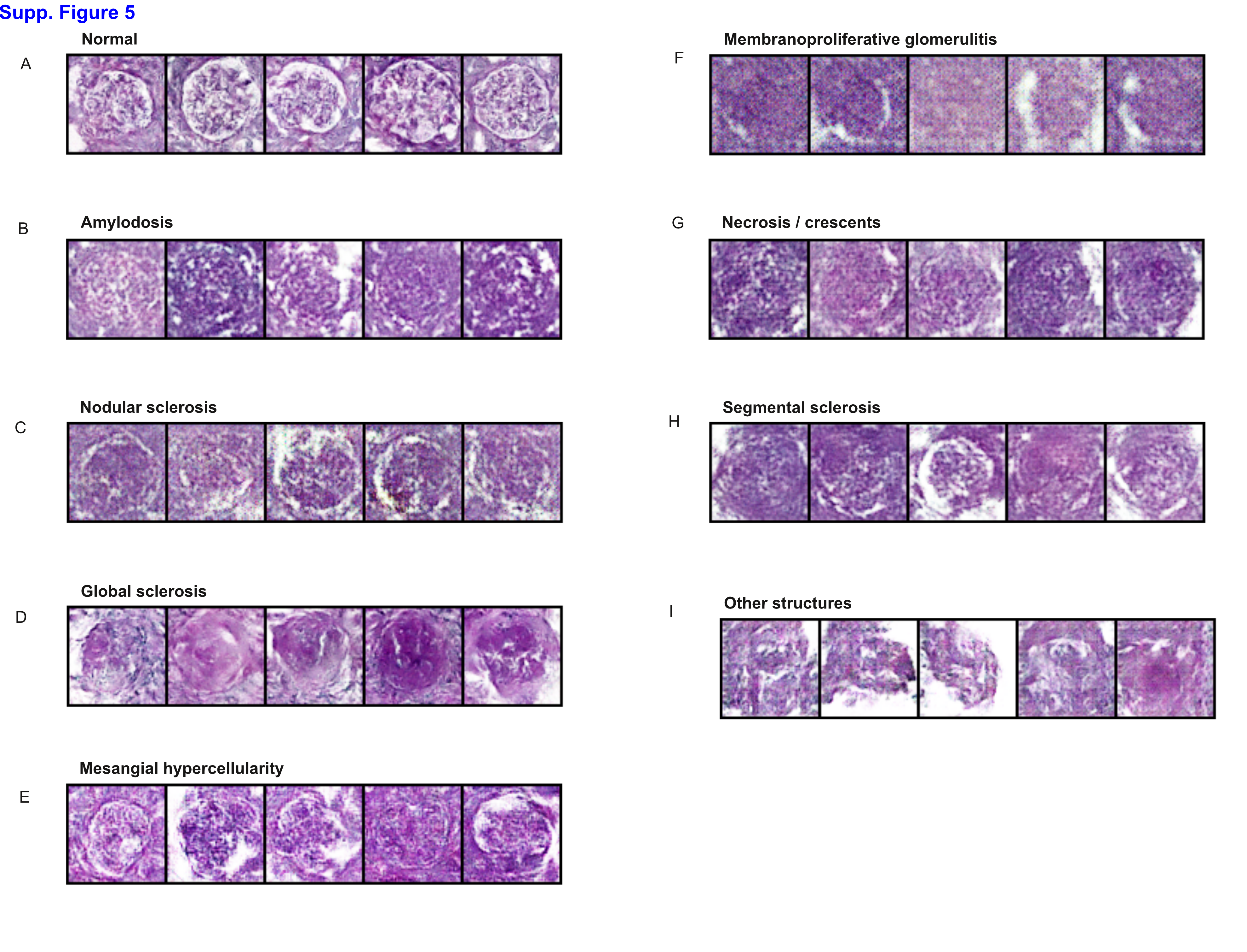

Supplement: Supplementary file 5 — Supp. Fig. 5 : Five examples of generative adversarial network results per defined pattern. Generative adversarial networks (GAN) combine two neural networks that can be used to produce example images from a trained CNN model. A-I show five example images trained for every class. (PNG 6398 kb) [file 40620_2021_1221_MOESM5_ESM.png]

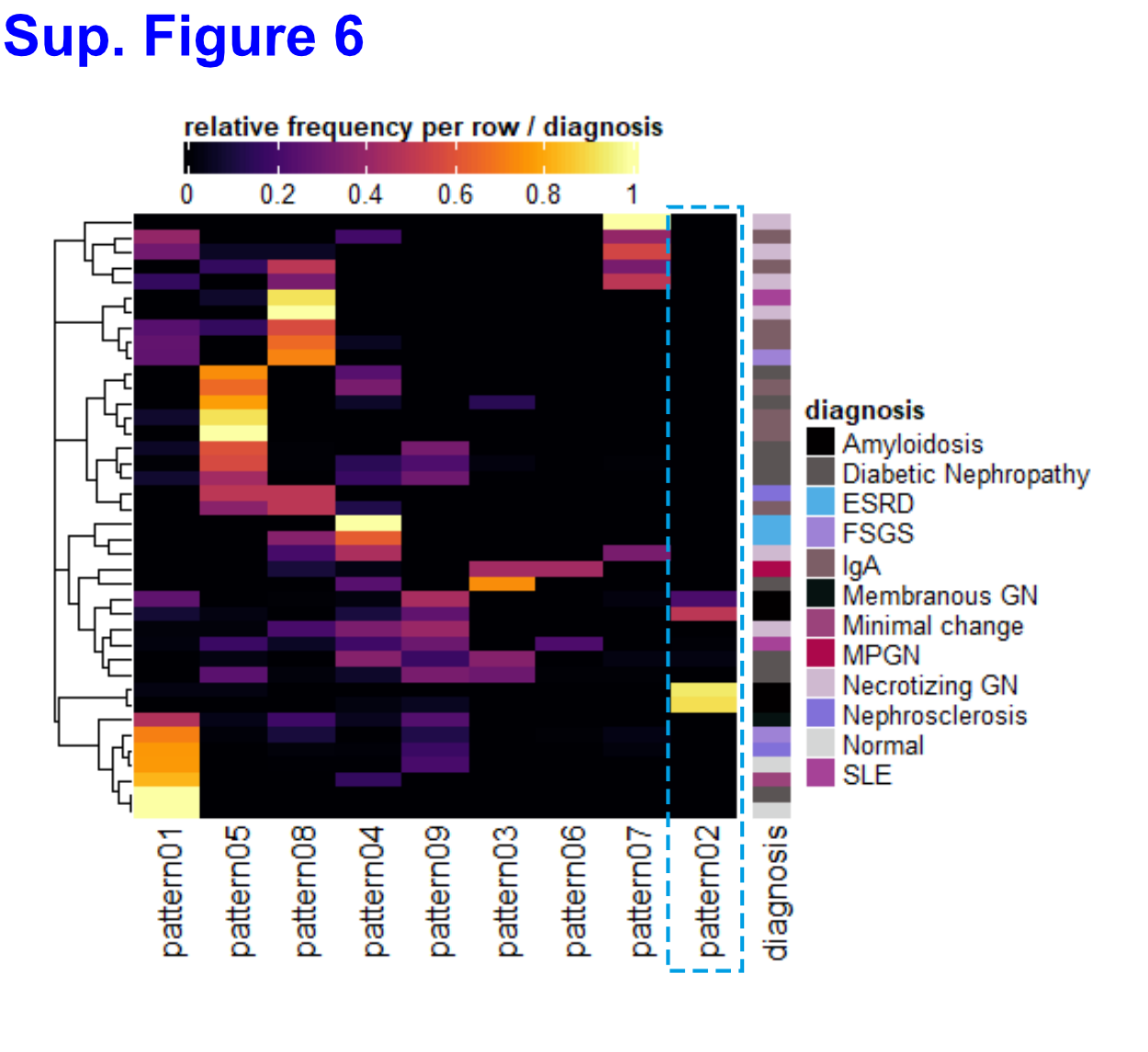

Supplement: Supplementary file 6 — Supp. Fig. 6 : Pattern distribution for a small heterogeneous cohort from routine diagnostics. For a small cohort composed of routine cases, all glomeruli per biopsy were cropped and subsequently analyzed. For these cases, a set of 12 diagnoses were defined: amyloidosis, diabetic nephropathy, ERSD (end-stage renal disease), FSGS (focal segmental glomerulosclerosis), IgA, membranous GN (glomerulonephritis), minimal change, MPGN (membranoproliferative glomerulonephritis), necrotizing GN (glomerulonephritis), nephrosclerosis, normal and SLE (systemic lupus erythematosus). The cases were arranged along the y-axis; thus, every row corresponds to one case. For every glomerulus, the main pattern was diagnosed by the ResNet152. The patterns are depicted on the x-axis. (PNG 217 kb) [file 40620_2021_1221_MOESM6_ESM.png]
